# Heredity

**Published:** 1896-04

**Authors:** W. T. Russell

**Affiliations:** Nashua, N. H.


### HEREDITY.[1]

BY W. T. RUSSELL, V.S.,
NASHUA, N. H.

I HAVE chosen for my subject the laws that govern heredity, and as they are constantly being brought to our notice in everyday life, the amount of debatable pabulum which the subject offers may, therefore, render it worthy as a subject for debate by this society. Before commencing I would disclaim the idea of putting forward anything new or original, and, further, would beg your indulgence for the shortcomings of a paper too hastily prepared. In treating the subject and in seeking analogies I have repeatedly gone to the human subject. With such increased opportunity for collecting statistics as man offers and the positive evidence which such at times affords, you will perhaps admit that this is allowable.

"Like begets like." At the outset I admit that I am a believer in this law to the bitter end. Its force is apparent in all forms of life constantly and everywhere. Both internally and externally do we find this, for not only features and conformation are reproduced, but bones and internal and circulatory organs are equally under its mighty domain. Practically, of course, this law is not exact, for, as Darwin puts it, "The number and diversity of inheritable deviations of structure, both

[1] Read before the New Hampshire Veterinary Medical Association, February 4, 1896.

those of slight and those of considerable physiological importance, are endless." Yet the law itself stands unchangeable. Of course, heredity has its opponents, but to ourselves naturally at once the art of breeding suggests itself as an argument of convincing weight in its favor.

Among men, heredity is to be noticed especially in nervous systems. Galton mentions that intellect and great ability are largely hereditary qualities. Musical attributes are common to families. Even trifling peculiarities are sometimes inherited; that of handwriting has often been observed.

The laws of heredity are generally summarized as four, viz.: Direct heredity, reversional heredity, indirect or collateral heredity, and the heredity of influence.

First we have direct heredity. This consists in the transmission of qualities supplied by both parents. This is obviously shown by the fusion of the characters of the parents, as exhibited by hybrids being distinct species, or strongly marked varieties among the lower animals, or as the offspring of parents belonging to two strongly contrasted races of men, such as white on the one hand and negro on the other. From a purely theoretical point of view, this law of direct heredity would be the common one, and, as described by Lucas, "It would consist in the absolute equilibrium of the moral and physical nature of the infant, of the integral resemblances of the two parents, or would equal the exact mean of the parents." As a fact, this rarely happens, the rule being that the influence of one or the other parent prevails in the offspring, and in this regard the tendency to cross-heredity, *i.e.*, from father to daughter or mother to son, appears to be the most frequent. Dr. Carpenter says: "It has long been a prevalent idea that certain parts of the organism of the offspring are derived from the male and certain other parts from the female parent; and although no universal rule can be laid down on this point, yet the observations that have been made by practical breeders seem to establish that this tendency has a real existence, the characters of the animal portion of the feline being especially (but not exclusively) derived from the male parent, and those of the organic apparatus being in like manner derived from the female parent. The former will be chiefly manifested in the external appearance, in the configuration of the head and limbs in the organs of the senses and in the locomotive apparatus; whilst the latter show themselves in the sizes of the body (which is primarily determined by the viscera contained in the trunk) and in the mode in which the vital functions are performed. But, however, general this rule may prove as regards the lower animals, it is by no means universal; for instances are far from infrequent in which the multiple progeny of one conception divide between them the characters of the parents in very different modes. Some litters of cross-bred pups are apt illustrations of this. It is rare to meet with instances in which some distinctive traits of both parents may not be traced in the offspring, these traits showing themselves in peculiarities of manner of gesture, in tendencies of thought or feeling, and in proneness to constitutional disorders. Let me offer as confirmatory evidence on this point the case of Leslie Geoffrey, an engineer in Mauritius, son of a white man and a stupid negress. Physically he was a negro, with the features and woolly hair of his mother's race, but in moral constitution he was so thoroughly white, as regarded intellectual development, that he was able to overcome the prejudice against him as a negro and attained the highest office in the island. Another fact that I would submit is that any peculiarity possessed in common by both parents is almost with certainty transmitted to the offspring. Equally certain is this rule in the case of disease-diatheses possessed by both parents; and Carpenter says, "The manifestation of it is likely to be yet more marked if the parents inherit the same tendencies."

Next we come to reversional heredity, wherein the offspring inherits the peculiarities of its grandparent or even more remote ancestor. Side by side with this we may take collateral or indirect heredity, which occurs between two persons and their ancestors in an indirect line, as between uncle and nephew, aunt and niece. Reversion is so common among animals that the term "throwing back" is of daily occurence. In this connection prepotency is often to be observed, one individual being especially powerful in transmitting his character through several generations. The laws which govern these variations are for the most part unknown. Darwin says: "No one can tell why the same peculiarity in different individuals of the same species, or in different species, is sometimes inherited and sometimes not so; why the child of ten reverts in certain characters to its grandfather or grandmother or more remote ancestor; why a peculiarity is often transmitted from one sex to both sexes or to one sex alone, more commonly but not exclusively to the like sex. It is a fact of some importance to us that peculiarities appearing in the males of our domestic animals are often transmitted exclusively, or in a much greater degree, to the males alone. A much more important rule which I think may be trusted is that, at whatever period of life a peculiarity first appears, it tends to reappear in the offspring at a corresponding age, though sometimes earlier. Hereditary diseases and some other facts make me believe that the rule has a wider extension, and that there is no apparent reason why a peculiarity should appear at a certain age. Yet, that it does tend to appear in the offspring at the same period at which it first appeared in the parent is an accepted fact.

Then, lastly, we have the heredity of influence, a form which offers many familiar examples and always affords interesting study. The phenomena in connection with it show that when a mother has previously borne offspring the influence of the father may be impressed on her progeny afterward begotten by a different parent. The well-known instance of a mare which having been impregnated by a zebra eventually transmitted zebra-marks to a succession of horse-begotten colts will readily occur to you. In the human family this is also true, as when a widow marrying a second time bears children with a strong resemblance to her first husband. A striking case is recorded of a negress whose first offspring was the child of a white man and ever afterward she bore half-caste children, although by a negro.

Perhaps, of all men, dog-breeders are as fully alive to the heredity of influence as any. The jealousy with which they guard the females previous to their first impregnation is a noteworthy tribute to the theory which it implies. It is the habit to attribute those cases to the mental impression produced by the first male parent upon the female, but a theory is also advanced which suggests that the blood of the female has imbibed from that of the fœtus, through the placental circulation, some of the attributes which the latter has derived from its male parent, and that the female may communicate these, with those proper to herself, to the subsequent offspring of a different male parentage.

We next have to consider some of the modifications of the foregoing laws as brought about by various influences, conditions, and surroundings for which domestication is solely responsible, for were there no domestication there might possibly have been no exception to heredity. Non-inheritance might be explained by the fact that strong hereditary tendencies, although existing, are overborne by unfavorable conditions of life; for instance, cart-horses will not transmit their fine size and great strength if living in cold, damp, or mountainous regions, and sheep in our tropical climates lose their wool in a few generations. Under this head, let me again quote Darwin, who says: "Under domestication we see much variability caused or at least excited by changed conditions of life, but often in so obscure a manner that we are tempted to consider the variations as spontaneous. Variability is governed by many complex laws, by correlated growth, compensation, the increased use and disuse of parts, and the definite action of surrounding conditions. As long as the conditions of life remain the same, we have reason to believe that a modification which has already been inherited for many generations may continue to be inherited for many generations to come. On the other hand, we have evidence that variability, where it has once come into play, does not cease under domestication for a very long period, nor do we know that it ever ceases, for new varieties are still occasionally produced by our oldest domesticated productions."

Before concluding this portion of my subject I cannot help referring to one other point. The fact that heredity is especially observable in nervous systems has already been referred to. Some of the deepest thinkers, in following up the subject with its connection with the evolution of the mind, gauge man's moral responsibility entirely by hereditary influences transmitted to him, and then apply the doctrine in consideration of the very complex question "freedom of will." In an extremely clever paper in the *Medical Service Monthly*, by Dr. Badran, to which I am indebted for some valuable notes, I find "The wicked are not wicked by deliberate choice of the advantages and pleasure of wickedness, but they are so in consequence of a warped inclination of their nature, which makes the evil seem good and the good seem evil." Criminals then are quite a manufactured article. There are no accidents in the laws of the universe; there is nothing accidental or supernatural in the impulse to do wrong. Both come by inheritance or education, or probably by a combination of the two. Science cannot believe that the impulses to do right are purely due to the grace of heaven and the impulses to do wrong to the malice of the evil one.

(To be concluded in the May number.)

